# Personality and Socioeconomic Predictors of Midlife Cognitive Functioning in Mexican-Origin Adults

**DOI:** 10.1093/geroni/igab046.038

**Published:** 2021-12-17

**Authors:** Katherine Lawson, Angelina Sutin, Richard Robins

**Affiliations:** 1 University of California, Davis, University of California, Davis, California, United States; 2 Florida State University College of Medicine, Tallahassee, Florida, United States; 3 University of California, Davis, Davis, California, United States

## Abstract

The present study aims to identify personality and socioeconomic (e.g., education, per capita income, economic stress) factors that contribute to midlife cognitive functioning across middle adulthood. Specifically, we examined how the growth trajectories of personality and socioeconomic factors across 12 years predict subsequent cognitive functioning, using data from a large sample of Mexican-origin adults (N=1,110; median age at Time 1=37 years, age range at Time 1=26-65). Personality was assessed using the Big Five Inventory, which assesses the Big Five domains as well as specific facets of each domain; economic stress was assessed using measures of negative economic events (e.g., job loss) and economic hardship (e.g., difficulty paying bills). Cognitive functioning was assessed using the NIH Cognitive Toolbox with measures of memory, language, and executive function. Findings from this work will help identify intervention targets for promoting healthy cognitive aging in midlife and beyond in Mexican-origin adults.

